# Aberrant corticostriatal functional circuits in adolescents with Internet addiction disorder

**DOI:** 10.3389/fnhum.2015.00356

**Published:** 2015-06-16

**Authors:** Fuchun Lin, Yan Zhou, Yasong Du, Zhimin Zhao, Lindi Qin, Jianrong Xu, Hao Lei

**Affiliations:** ^1^National Center for Magnetic Resonance in Wuhan, State Key Laboratory of Magnetic Resonance and Atomic and Molecular Physics, Wuhan Institute of Physics and Mathematics, Chinese Academy of SciencesWuhan, China; ^2^Department of Radiology, RenJi Hospital, Jiao Tong University Medical SchoolShanghai, China; ^3^Department of Child and Adolescent Psychiatry, Shanghai Mental Health Center, Jiao Tong UniversityShanghai, China

**Keywords:** corticostriatal circuits, functional connectivity, internet addiction disorder, neuropsychological measures, resting-state fMRI

## Abstract

Abnormal structure and function in the striatum and prefrontal cortex (PFC) have been revealed in Internet addiction disorder (IAD). However, little is known about alterations of corticostriatal functional circuits in IAD. The aim of this study was to investigate the integrity of corticostriatal functional circuits and their relations to neuropsychological measures in IAD by resting-state functional connectivity (FC). Fourteen IAD adolescents and 15 healthy controls underwent resting-state fMRI scans. Using six predefined bilateral striatal regions-of-interest, voxel-wise correlation maps were computed and compared between groups. Relationships between alterations of corticostriatal connectivity and clinical measurements were examined in the IAD group. Compared to controls, IAD subjects exhibited reduced connectivity between the inferior ventral striatum and bilateral caudate head, subgenual anterior cingulate cortex (ACC), and posterior cingulate cortex, and between the superior ventral striatum and bilateral dorsal/rostral ACC, ventral anterior thalamus, and putamen/pallidum/insula/inferior frontal gyrus (IFG), and between the dorsal caudate and dorsal/rostral ACC, thalamus, and IFG, and between the left ventral rostral putamen and right IFG. IAD subjects also showed increased connectivity between the left dorsal caudal putamen and bilateral caudal cigulate motor area. Moreover, altered cotricostriatal functional circuits were significantly correlated with neuropsychological measures. This study directly provides evidence that IAD is associated with alterations of corticostriatal functional circuits involved in the affective and motivation processing, and cognitive control. These findings emphasize that functional connections in the corticostriatal circuits are modulated by affective/motivational/cognitive states and further suggest that IAD may have abnormalities of such modulation in this network.

## Introduction

Internet addiction disorder (IAD), a prevalent mental health concern around the world, has attracted considerable attention from the public and scientific community (Spada, 2014). In the appendix of the newly released Diagnostic and Statistical Manual of Mental Disorders Edition, fifth edition (DSM-5), Internet gaming disorder, a major subtype of IAD, is listed as a disorder requiring further study (Petry et al., 2014). IAD lead to negative consequences in daily life; however, little is known about the biomarkers, prevalence, course and treatment outcomes associated with IAD.

To understand the neurobiological mechanisms underlying IAD, imaging studies have been performed to investigate structural and functional abnormalities associated with IAD. Brain structural and functional changes associated with IAD have been reviewed in previous studies elsewhere (Kuss and Griffiths, 2012; Ko et al., 2015; Lin and Lei, 2015). In brief, it is consistently shown that the prefrontal cortex (PFC) and striatum are implicated in IAD. Subjects with IAD have reduced gray matter densities/volumes (Yuan et al., 2011; Zhou et al., 2011; Weng et al., 2013), cortical thickness (Hong et al., 2013a; Yuan et al., 2013), glucose metabolism (Tian et al., 2014) and altered brain activation (Dong et al., 2013a; Ko et al., 2014) in the PFC including the dorsolateral PFC, orbitofrontal cortex (OFC) and anterior cinguate cortex (ACC). IAD addicts were also found to have lower level of dopamine D2 receptors (Kim et al., 2011; Hou et al., 2012), altered glucose metabolism (Park et al., 2010a) and brain activation (Ko et al., 2014; Li et al., 2014) in the striatum. These findings are in line with current pathophysiological model emphasizing the prominent role to the striatum and PFC in addiction disorders (Goldstein and Volkow, 2011; Limbrick-Oldfield et al., 2013).

Resting-state functional connectivity (FC), measuring inter-regional correlations of spontaneous brain activity from blood oxygen level dependent (BOLD) functional magnetic resonance imaging (MRI) signals, has been widely used to investigate functional organization/connectivity of the brain. With this technique, evidence suggests that corticostriatal functional circuits are critical to the emergence of repetitive and compulsive behaviors, habitual behavior, reward-seeking and novelty-seeking behaviors, and addictive behavior (Feil et al., 2010; Shepherd, 2013). Moreover, altered corticostriatal functional circuits were found in autism (Di Martino et al., 2011), obsessive-compulsive disorder (Harrison et al., 2009; Posner et al., 2014; Burguière et al., 2015), and major depression disorder (Furman et al., 2011). Disrupted corticostriatal network has also been reported in frequent pornography users who were involved in reward-related and addictive behaviors (Kühn and Gallinat, 2014). Imaging studies have also demonstrated strong links between substance use disorders and dysfunction within the corticostriatal functional circuits (Feil et al., 2010; Volkow et al., 2013).

Anatomically, striatum is a heterogeneous structure that can be parceled into subregions, which is involved in functionally segregated corticostriatal circuits underpinning different cognitive functions (Alexander et al., 1986; Choi et al., 2012; Gordon et al., 2015; Manza et al., 2015). For example, by parceling caudate and putamen into three regions, respectively, Di Martino et al. (2008) delineated the detailed patterns of corticostriatal functional circuits which are involved in affective, motivational, cognitive and motor processes (Di Martino et al., 2008). It has been shown by previous studies that functional/effectivity connectivity between the striatum and cortex is reduced in IAD subjects (Hong et al., 2013b, 2015; Li et al., 2014; Wee et al., 2014). However, most of these studies did not investigate into how the functionally segregated corticostriatal circuits specific to the striatum subregions are affected.

Therefore, in the present study, we used a validated set of six bilateral striatal seeds (three seeds in the caudate and three seeds in the putamen) to explore alternations of specific corticostriatal functional circuits in adolescents with IAD. The aims are: (1) to investigate differences in the topographic distribution of corticostriatal functional circuits between adolescents with IAD and healthy controls without IAD; and (2) to illuminate relationships between corticostriatal functional circuits and neuropsychological measures in IAD subjects.

## Materials and Methods

### Subjects

The study was approved by the Ethics Committee of RenJi Hospital of Shanghai Jiao Tong University Medical School. The participants and their parents provided written informed consent before MRI examinations.

Eighteen right-handed adolescents with IAD and 18 right-handed, age-, gender-, and education-matched healthy controls participated in this study. The diagnosis standard for IAD was established by the modified Young’s diagnostic questionnaire for Internet addiction criteria by Beard and Wolf (Beard and Wolf, 2001). All subjects were screened for psychiatric disorders with the Mini International Neuropsychiatric Interview for Children and Adolescents (MINI-KID; Sheehan et al., 2010). The exclusion criteria included a history of substance abuse or dependence; a history of major psychiatric disorders, such as schizophrenia, depression, anxiety disorder, psychotic episodes, or hospitalization for psychiatric disorders. The IAD subjects received no medication treatments while a small number of IAD individuals received psychotherapy. The structural and diffusion MRI data of these subjects had been used in our previous studies (Zhou et al., 2011; Lin et al., 2012). For this study, the rs-fMRI data from three controls and four IAD subject were discarded due to large head motion (see the Preprocessing Section). As a result, a total of fifteen controls and fourteen IAD subjects were used in the study. Detailed demographic information for all subjects is listed in Table 1.

**Table 1:** **Demographic and behavioral characteristics of the subjects used in this study**.

	CON (*n* = 15)	IAD (*n* = 14)	*p* value
	(Mean ± SD)	(Mean ± SD)
Age	17.87 ± 2.52	17.12 ± 2.73	0.45
Gender (M/F)	13/2	12/2	0.94
Education (years)	11.60 ± 3.07	10.57 ± 2.62	0.34
Young’s Internet Addiction Scale	36.17 ± 10.66	65.07 ± 13.25	**<0.0001**
Time Management Disposition Scale	125.43 ± 19.60	122.14 ± 23.42	0.69
Strength and Difficulties Questionnaire	16.57 ± 3.96	22.71 ± 2.55	**<0.0001**
Barratt Impulsiveness Scale-11	67.21 ± 8.13	70.07 ± 13.56	0.51
The Screen for Child Anxiety Related Emotional Disorders	24.46 ± 6.33	38.29 ± 10.77	**<0.001**
Family Assessment Device	117.29 ± 11.16	129.21 ± 13.55	**0.017**

*Abbreviation: CON, controls; IAD, Internet addiction disorder; SD, standard deviation.Two-sample t test was used for group comparisons but chi-square was used for gender comparison*.

### Neuropsychological Assessments

Six questionnaires, including the Young’s Internet Addiction Scale (YIAS; Young, 1996), Strengths and Difficulties Questionnaire (SDQ; Goodman, 1997), Time Management Disposition Scale (TMDS; Huang and Zhang, 2001), Barratt Impulsiveness Scale-11 (BIS; Patton et al., 1995), the Screen for Child Anxiety Related Emotional Disorders (SCARED; Birmaher et al., 1997) and Family Assessment Device (FAD; Epstein et al., 1983), were used to evaluate the participants’ neuropsychological features.

### Image Acquisition

Resting-state fMRI scans were performed by an echo-planar imaging on a 3.0 Tesla Phillips Achieva medical scanner with the following parameters: repetition time = 2000 ms; echo time = 30 ms; flip angle = 90°; acquisition matrix = 64 × 64; field of view = 230 × 230 mm^2^; slice thickness = 4 mm with no gap. Each brain volume comprised of 34 axial slices and each run contained 220 volumes. During the data acquisition, all subjects were instructed to rest, keep their eyes closed, and not think of anything in particular.

### Data Preprocessing

Data preprocessing was performed using by SPM8.^1^ The first 10 volumes for each subject were discarded to avoid the effects of system instability. The remaining 210 volumes were corrected for the acquisition time delay and realigned to the first volume. Subjects with maximum displacement in any direction of larger than 2.0 mm or head rotation of larger than 2.0° were excluded from this study. As a result, data of four IAD subjects and three controls were excluded. The results showed there were no differences on head motion between two groups (*p* = 0.55 for translational motion and *p* = 0.43 for rotational motion). The realigned images were then spatially normalized to the Montreal Neurological Institute space and re-sampled to a 3 mm isotropic voxel. The normalized images were smoothed with a 6-mm full width at half maximum isotropic Gaussian kernel and several sources of spurious variances including the head-motion parameters, linear drift, global BOLD signals, and BOLD signals in white matter and cerebro-spinal fluid were removed through linear regression. Finally, temporal band-pass filtering (0.01–0.08 Hz) was performed on the time series of each voxel using an ideal rectangle window.

### Functional Connectivity Analysis

We employed six previously validated bilateral striatal regions of interest (“seeds”; Di Martino et al., 2008). Caudate seeds included the inferior ventral striatum (VSi, corresponding to the nucleus accumbens; ±9, 9, −8), superior ventral striatum (VSs; ±10, 15, 0) and dorsal caudate (DC; ±13, 15, 9). Putamen seeds included the ventral rostral putamen (VRP; ±20, 12, −3), dorsal rostral putamen (DRP; ±25, 8, 6), and dorsal caudal putamen (DCP; ±28, 1, 3). The radius for each seed is 6 mm. The coordinates for right and left hemisphere seeds were defined in the MNI space. These seeds were validated based on anatomical and functional subdivisions of the striatum, and their connectivity patterns have been replicated independently (Di Martino et al., 2008, 2011; Harrison et al., 2009; Kelly et al., 2009; Choi et al., 2012; Gabbay et al., 2013; Gordon et al., 2015; Manza et al., 2015).

For each subject, a cross-correlation coefficient map for each seed was first obtained by calculating the cross-correlation coefficient between the average time courses of the seed subregion and that of each voxel of the whole brain through regressing the effects of head motion, linear drift, and brain activity from cerebrospinal fluid and white matter. And then the cross-correlation coefficient map was converted to *z*-value maps by Fisher’s r-to-z transformation to approach a normal distribution. The *z*-values maps were entered into a voxel-wise one-sample *t* test to determine group FC maps with height (*p* < 0.001) and extent (*p* < 0.001) thresholds corrected at the whole-brain level (Greicius et al., 2007). Group FC maps from both IAD subjects and healthy controls were combined by using an “OR” operation to generate a combined mask, which was used to constrain the subsequent group-between analyses. Then the *z*-value maps within this mask were entered into a voxel-wise two-sample *t* test with age and gender as covariates to evaluate group-between FC differences. The combined threshold of *p* < 0.005 for each voxel and cluster size of 351–405 mm^3^ (left (l) VSi: 351 mm^3^; right (r) VSi: 378 mm^3^; lVSs: 405 mm^3^; rVSs: 378 mm^3^; lDC: 405 mm^3^; rDC: 405 mm^3^; lDRP: 378 mm^3^; rDRP: 405 mm^3^; lDCP: 405 mm^3^; rDCP: 432 mm^3^; lVRP: 405 mm^3^; rVRP: 405 mm^3^), corresponding to a corrected *p* < 0.05 was used to obtain the significant group-between FC difference maps. This correction was confined within the combined mask and was determined by 5000 Monte Carlo simulations using the AFNI AlphaSim program.^2^

### Brain-behavior Associations

Step-wise multiple regression analyses with averaged FC strength in the regions showing group-between FC differences as dependent variable and age, gender, education, YIAS, SDQ, SCARED, FAD, TMDS, and BIS as independent variables was performed to check whether the changed functional circuits are correlated with the behavioral scores.

## Results

### Demographic and Behavioral Measures

Participants in IAD group and normal control group were matched on age, gender and years of education. There were no significant differences in the TMDS and BIS between the two groups while IAD subjects had higher YIAS (*p* < 0.0001), SDQ (*p* < 0.0001), SCARED (*p* < 0.001) and FAD (*p* = 0.017) scores than the controls. The demographic characteristics and behavioral measures for IAD and control subjects were listed in Table 1.

### Corticostriatal Functional Circuits

**Figure 1:**
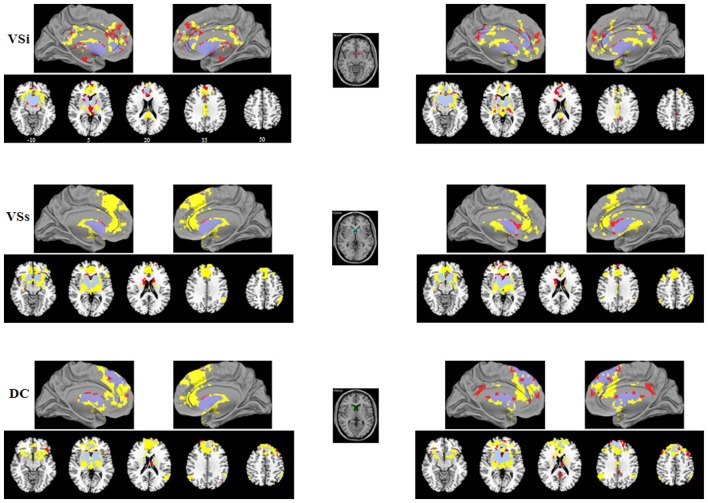
**Functional connectivity (FC) maps of the caudate seeds for each group**. FC Maps for Internet addiction disorder (IAD) adolescents (red) and normal controls (HC; yellow) were generated separately and then overlaid together for display purposes; light purple color indicates overlapped areas for both groups. The left (right) column indicates the FC maps generated by left (right) caudate seeds. The middle column indicates the caudate seeds. The left side of the image corresponds to the left hemisphere of the brain. VSi, inferior ventral striatum; VSs, superior ventral striatum; DC, dorsal caudate.

**Figure 2:**
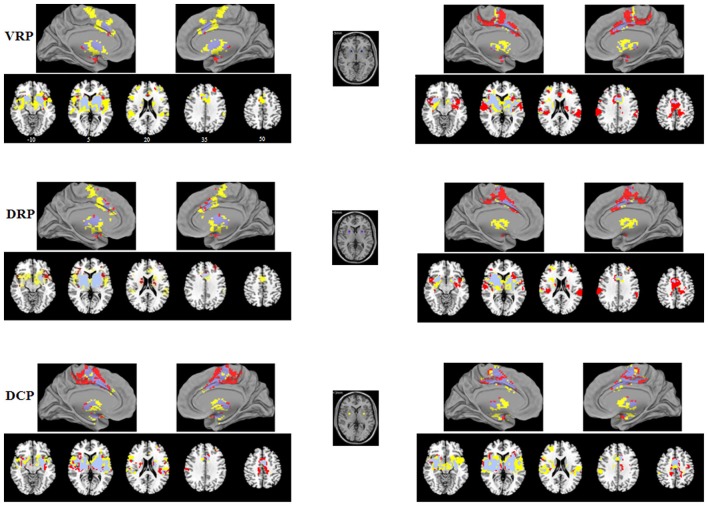
**Functional connectivity (FC) maps of the putamen seeds for each group**. FC maps for IAD adolescents (red) and normal controls (HC; yellow) were generated separately and then overlaid together for display purposes; light purple color indicates overlapped areas for both groups. The left (right) column indicates the FC maps generated by left (right) putamen seeds. The middle column indicates the putamen seeds. The left side of the image corresponds to the left hemisphere of the brain. VRP, ventral rostral putamen; DRP, dorsal rostral putamen; DCP, dorsal caudal putamen.

**Figure 3:**
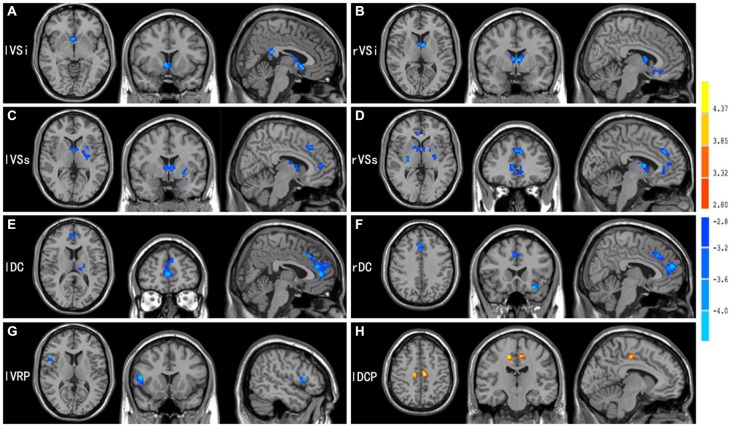
**Brain areas showed significant FC differences between adolescents with IAD and matched normal controls (*p* < 0.05, AlphaSim corrected), when seed regions were located in the (A) lVSi, (B) rVSi, (C) lVSs, (D) rVSs, (E) lDC, (F) rDC, (G) lVRP, and (H) lDCP. For further details, see Table 2**. Hot and cold colors indicate FC increases and decreases in IAD when compared with controls.

Consistent with prior works, seed-based FC analyses provided detailed maps of distinct functional circuits for each of the six seeds in the striatum per hemisphere. FC pattern for the caudate and putamen seeds were shown in Figures 1, 2, respectively. Our findings recapitulated previous studies (Di Martino et al., 2008, 2011; Harrison et al., 2009; Kelly et al., 2009; Choi et al., 2012; Gabbay et al., 2013; Gordon et al., 2015; Manza et al., 2015) and were consistent with known anatomical connectivity (Haber, 2003) and functional activation adapted from a meta-analysis of the tasked literature (Postuma and Dagher, 2006). Although the striatal FC patterns for IAD subjects and normal controls were similar for each of the six striatal seeds, the extents of the IAD group were reduced when compared with those of control group. Specific findings are exhibited in Figure 3; Table 2 and are described below.

**Table 2:** **Regions showing significant functional connectivity differences between adolescents with Internet addiction disorder (IAD) and matched control subjects (*p* < 0.05, AlphaSim corrected)**.

Seed	Seed	Regions of differences	Peak coordinates (MNI)			Peak *T*	Cluster size (voxels)
			*X*	*Y*	*Z*
Caudate	L VSi	Bilateral caudate head, subgenual ACC	6	6	0	−4.64	101
		Bilateral ventral PCC	6	−42	18	−4.34	33
	R VSi	Bilateral caudate head, subgenual ACC	−3	0	6	−5.79	99
	L VSs	Left putamen, pallidum, insula, IFG	−30	9	−3	−5.95	115
		Bilateral dorsal ACC	3	24	42	−4.89	78
		Bilateral ventroanterior thalamus	−6	−9	12	−4.71	74
		Bilateral rostral ACC	6	45	6	−4.25	35
	R VSs	Bilateral dorsal caudate, ventroanterior thalamus	−9	0	9	−5.84	112
		Right putamen/lentiform nucleus	30	−15	0	−5.00	35
		Bilateral dorsal ACC	0	36	33	−4.90	97
		Bilateral rostral ACC	9	33	9	−4.58	113
		Left putamen, pallidum, insula, IFG	−33	21	−9	−4.08	105
	L DC	Left ventrolateral thalamus	−18	−9	3	−4.40	36
		Right dorsal/rostral ACC	3	39	18	−4.34	251
	R DC	Right dorsal/rostral ACC	9	54	18	−4.75	114
		Left IFG	−27	24	−18	−4.70	31
		Bilateral dorsal ACC	−12	30	33	−4.56	112
Putamen	L DCP	Right caudal cigulate motor are	15	−21	45	4.90	42
		Left caudal cigulate motor are	−6	−21	48	4.57	26
	L VRP	Right IFG	51	15	12	−5.76	36

*Note. Negative T values mean the functional connectivity of IAD is reduced while positive T values mean the functional connectivity of IAD is increased. Abbreviation: MNI, Montreal Neurological Institute; ACC, anterior cingulate cortex; PCC, posterior cingulate cortex; IFG, inferior frontal gyrus; L, left; R, right; VSi, inferior ventral striatum; VSs, superior ventral striatum; DC, dorsal caudate; DCP, dorsal caudal putamen; VRP, ventral rostral putamen*.

### Inferior and Superior Ventral Striatum

Both groups displayed an FC gradient from ventromedial to dorsolateral divisions of prefrontal and ACC going from VSi to VSs. Additionally, the VSi showed significant positive correlation with the posterior cingulated cortex (PCC). When FC maps were compared between groups, significant differences were observed for the VSi and VSs. With regard to the VSi, IAD adolescents demonstrated significantly reduced FC with the caudate head and subcallosal ACC bilaterally. Decreased FC was also found between the left VSi and the PCC bilaterally. For the VSs seed region, IAD subjects exhibited lower FC with dorsal/rostral ACC and ventral anterior thalamus bilaterally, and the left subcortical areas including the putamen, pallidum, insula and inferior frontal gyrus (IFG).

### Dorsal Caudate

In both IAD subjects and healthy controls, the DC showed positive relationships with brain regions involved in cognitive control. Direct group comparisons revealed that IAD displayed decreased FC between the DC and dorsal/rostral ACC bilaterally. The left DC also showed reduced FC with the left ventral lateral thalamus, as well as the right DC displayed lower positive relationships with the left IFG in IAD.

### Dorsal Caudal and Dorsal Rostral Putamen

Consistent with their role in motor control, the dorsal putamen seeds exhibited significant positive relationships with the primary and secondary sensorimotor areas for both IAD and healthy subjects. However, relative to healthy controls, IAD showed increased FC between the left DCP and caudal cingulate motor area bilaterally.

### Ventral Rostral Putamen

The VRP seed positively correlated with the rostral ACC and dorsal lateral PFC commonly associated with conflict monitoring and error related processes. Although IAD showed less extender FC with other brain region, only FC between the left VRP and right IFG demonstrated significant differences between groups.

### Brain-behavior Associations in IAD

**Figure 4:**
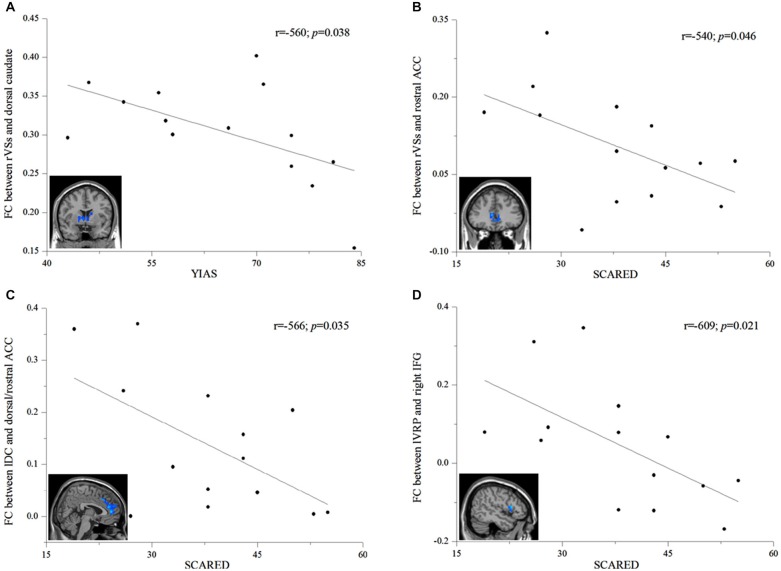
**Correlation analysis between FC strength and behavioral measures within the IAD group. (A)** Correlations between FC strength (indicated by average *z* value) of the right superior ventral striatum (rVSs) to the dorsal caudate and the Young’s Internet addiction scale (YIAS; *r* = −0.560, *p* = 0.038). **(B)** Correlations between the FC strength (indicated by average *z* value) of the rVSs to the rostral anterior cinguate cortex (ACC) and the screen for child anxiety related emotional disorders (SCARED; *r* = −0.540, *p* = 0.046). **(C)** Correlations between the FC strength (indicated by average *z* value) of the left dorsal caudate (lDC) to the rostral/dorsal ACC and the SCARED; (*r* = −0.566, *p* = 0.035). **(D)** Correlations between the FC strength (indicated by average *z* value) of the left ventral rostral putamen (lVRP) to the right inferior frontal gyrus (IFG) and the SCARED (*r* = −0.609, *p* = 0.021).

In IAD subjects, higher scores on the YIAS predicted lower FC strength between the right VSs and the bilateral dorsal caudate (*r* = −0.560; *p* = 0.038; Figure 4A). Moreover, higher SCARED scores predicted lower FC strength between the right VSs and the bilateral rostral ACC (*r* = −0.540; *p* = 0.046; Figure 4B), between the left DC and the bilateral dorsal/rostral ACC (*r* = −0.566; *p* = 0.035; Figure 4C), and between the left VRP and the right IFG (*r* = −0.609; *p* = 0.021; Figure 4D). We also used spearman correlation to detect associations between the changed FC and behavior measures. The results of spearman regression were similar with those of linear regression. YIAS was correlated with FC strength between the right VSs and the bilateral dorsal caudate (*r* = −0.594; *p* = 0.025). The SCARED scores were associated with FC strength between the right VSs and the bilateral rostral ACC (*r* = −0.548; *p* = 0.042), and between the left VRP and the right IFG (*r* = −0.666; *p* = 0.009). The SCARED scores had a trend correlation with FC strength between the left DC and the bilateral dorsal/rostral ACC (*r* = −0.464; *p* = 0.095).

## Discussion

To our knowledge, this is the first study to investigate the integrity of the corticostriatal functional networks and relationships between circuit-level abnormalities and clinical measures in IAD. For both IAD subjects and controls, we replicated the findings of Di Martino et al. (2008), observing patterns of connectivity consistent with hypothesized affective and motivation (inferior ventral striatum), cognitive (ventral putamen, dorsal caudate, superior ventral striatum) and motor (dorsal putamen) subdivisions of the striatum. When compared with controls, IAD show similar connectivity patterns but altered connectivity strengths for every striatum subregion except the DRP. Moreover, we found YIAS scores was negatively related with connectivity strength between the right VSs and the dorsal caudate bilaterally, and SCARED scores was inversely associated with connectivity strengths between the right VSs and the bilateral rostral ACC, between the left DC and the bilateral dorsal/rostral ACC as well as between the left VRP and the right IFG. These relationships indicates that more severe the Internet addiction, the weaker the connectivity strengths between these regions. Our findings suggest that corticostriatal functional circuits may be used as a qualified biomarker to understand the underlying neural mechanisms of injury or to evaluate the effectiveness of specific early interventions in IAD.

### Disrupted Corticostriatal Functional Circuits in IAD

In the current study, the VSi seed showed decreased connectivity with the caudate head, subgenual ACC and PCC in the IAD group, a connection known to be important for affective and motivational processing (Johansen-Berg et al., 2008; Beckmann et al., 2009). The finding of reduced connectivity between the nucleus accumbens/VSi and caudate head implies the changed reward-related functions in IAD, indicating Internet addicts may prefer to select smaller immediate rewards (i.e., immediate euphoric effects) rather than larger rewards that occur in the future, such as good health, good relationships or occupational success (Irvine et al., 2013). As noted, decreased activation in the caudate after continuous wins was observed in IAD (Dong et al., 2013b). Subgenual ACC, a high probability of connectivity with the nucleus accumbens/VSi, is a critical hub within distributed networks in charge of negative emotional arouse or regulation (Johansen-Berg et al., 2008; Rudebeck et al., 2014). Previous brain imaging studies showed that subgenual ACC is implicated in experience of negative mood states (Mayberg et al., 1999) and subgenual ACC is a target with deep brain stimulation for treatment of depression (Liston et al., 2014). Neuropsychological studies revealed that high rates of anxiety and mood disorders were found in subjects with IAD (Bozkurt et al., 2013; Zhang et al., 2013). The PCC, a central brain region of the default mode network, is implicated in self-referential functions (Vogt et al., 2006). Abnormal gray matter density (Zhou et al., 2011) and white matter microstructure (Dong et al., 2012a) in the PCC were reported in IAD individuals. The clinical psychological studies also found that Internet addiction university students have lower self-directedness and cooperativeness scores (Dalbudak et al., 2013a), suggesting that IAD subjects have lower degree for self-referential functions. Taken together, the findings of reduced connectivities between the VSi and the caudate head, subgenual ACC and PCC indicate that IAD adolescents show abnormal affective and motivational processing.

Our finding of decreased connectivity between the caudate (VSs and DC) and the bilateral dorsal/rostral ACC imply dysfunction of the croticostriatal-limbic circuitry involved in cognitive and emotional control (Botvinick et al., 2004; Li and Sinha, 2008) in IAD. Dorsal ACC has been associated with maintenance of working memory, conflict monitoring and error processing as well as rostral ACC is involved in affective processing and emotional regulation (Bush et al., 2000). As noted, lower gray matter density in the left dorsal ACC was found in the cohort of structural MRI data in our previous studies (Zhou et al., 2011). Another research showed that IAD had a decreased gray matter volume in the rostral ACC (Yuan et al., 2011). Greater activity in the ACC was also revealed for the interference condition of the stroop paradigm (Dong et al., 2012b) and a meta-analysis showed that IAD had a significant hyperactivation in medial frontal/ACC (Meng et al., 2014). Subjects with IAD also demonstrated an impaired error monitoring ability compared to controls, which was related with the stronger activity in dorsal ACC in error responses (Dong et al., 2013a). Behavior studies showed that IAD individuals were associated with longer reaction time and more response errors in incongruent conditions than the controls without IAD (Dong et al., 2011). Reduced connectivity between VSs and insula was also reported in IAD. The insula has been previously shown to be consistently activated during performance monitoring, and modulated by error awareness (Menon and Uddin, 2010). A meta-analysis study of brain imaging suggested that the insula is engaged in error awareness (Klein et al., 2007). Thus, the insula play an important role in error processing in terms of adjusting the human behavior. As noted, IAD subjects exhibited lower gray matter density (Zhou et al., 2011) and decreased cortical thickness (Yuan et al., 2013) in the insula. Moreover, decreased insular activation during error processing has been previously found in subjects with IAD (Ko et al., 2014). Therefore, like substance dependence, disrupted cognitive control and emotional stress processing along with compulsive Internet use constitute the core of croticostriatal-limbic functional deficits in IAD addicts.

IAD also demonstrated decreased connectivity between the striatum (VSs, DC and VRP) and the IFG, a connection known to be involved in inhibitory control (Chambers et al., 2009; Swick et al., 2011). Deficits in inhibitory control may contribute to loss of control over their Internet use and persistence in online gaming use despite personal distress, symptoms of psychological dependence, and diverse negative consequences. Epidemiological studies showed that adolescents with IAD exhibited more impulsivity (i.e., deficits in response inhibition) than controls without IAD (Cao et al., 2007; Dalbudak et al., 2013b). One neuropsychological study displayed impaired response inhibition in subjects with IAD (Zhou et al., 2012). Another event-related brain potentials with the Go/No-Go task study demonstrated that the IAD students had less efficiency in information processing and lower impulse control than their normal peers (Dong et al., 2010). Moreover, the subjects with Internet gaming disorder exhibited higher brain activation when processing response inhibition over the left frontal lobe than controls (Ko et al., 2014). Reduced connectivities between striatum (VSs and DC) and pallidum and thalamus were also found in the IAD group. In the corticostriatal circuits, the pallidum is the output of the striatum and the pallidum connects to the thalamus which projects to the cortex (Alexander et al., 1986). These circuits are thought to be important for focusing and maintaining desired behaviors while suppressing unwanted behaviors (Haber and McFarland, 2001). IAD individuals are known to have difficulties with response inhibition, which likely contributes to their propensity to relapse in the presence of Internet related cues. Therefore, the findings implicate that poor inhibitory control, a decreased ability to suppress automatic and habitual behaviors, is prevalent in subjects with IAD.

Interestingly, IAD showed increased connectivity between the left DCP and the bilateral caudal cigulate motor areas which are often activated during simple arm movements (Shima and Tanji, 1998). Given Internet addicts spend a tremendous amount of time online and become astonishingly skilled and accurate in mouse clicking and keyboard typing (Kuss and Griffiths, 2012), it is possible that that such training processes may induce neuroplastic changes in the caudal cigulate motor related areas.

### Relationships Between Corticostriatal Functional Circuits and Behavior in IAD

In this study, we investigated the behavioral correlates of alterations of corticostriatal functional circuits in IAD adolescents. Reduction of FC strength between the right VSs and the bilateral dorsal caudate of the IAD subjects correlated significantly with increase of YIAS score; while higher SCARED score appeared to be related with lower FC strengths between the right VSs and the bilateral rostral ACC, between the left DC and the bilateral dorsal/rostral ACC, and between the left VRP and the right IFG. The YIAS is a widely used questionnaire for evaluating the dependence of the Internet. Previous psychometric studies reported that IAD subjects had higher YIAS scores than those without IAD (Cao and Su, 2007). Since reduced connectivity is thought to indicate more difficulty in engaging a circuit when needed, this observation of the negative correlation between YIAS scores and connectivity strength between the right VSs and the bilateral dorsal caudate implied that IAD subjects with higher YIAS scores appeared to seek out the supraphysiological stimulation of Internet over natural rewards. The SCARED is a reliable and valid self-report questionnaire that measures symptoms of anxiety disorders in children (Birmaher et al., 1997). Neuropsychological studies revealed that IAD adolescents had significantly higher SCARED score than those without IAD (Xiuqin et al., 2010). The negative association between SCARED scores and connectivity strengths arise from dysfunction of corticostriatal circuits which are involved in affection regulation. Moreover, the findings of significant associations between connectivity strength within the corticostriatal circuits and behavioral features indicate that the corticostriatal networks may serve as a predictor of abstinence or a potential new treatment target for IAD.

### Comparisons with Corticostriatal Functional Circuits Abnormalities in Drug Addiction

Resting-state FC studies have also demonstrated strong associations between drug addiction and corticostriatal functional circuits abnormalities. For example, increased FC was observed between the left ventral striatum and right OFC, extending into rostroventral ACC in cocaine addiction (Wilcox et al., 2011). The FC strength within the striatal-dorsolateral PFC was positively correlated with the amount of cocaine use in the cocaine users, and the balance between striatal-dorsal ACC and striatal-anterior prefrontal/orbitofrontal cortex circuits was significantly associated with loss of control over cocaine use (Hu et al., 2015). Chronic alcohol abuse also has a deleterious effect on the function within the corticostriatal circuits. For instance, the dorsal striatum-mOFC FC was impaired (Lee et al., 2013) and the impaired frontostriatal connectivity induced abnormal decision-making and reward and response inhibition in alcohol dependence (Park et al., 2010b; Courtney et al., 2013; Forbes et al., 2014). As for nicotine addiction, the decreased FC between ventral striatum and dorsal anterior cingulated cortex negatively correlated with nicotine dependence severity (Hong et al., 2009). Moreover, increased FC between the nucleus accumbens and ventral/rostral ACC and OFC, between the right caudate and bilateral middle frontal gyrus and right superior frontal gyrus has been observed in chronic heroin users (Ma et al., 2010; Wang et al., 2013). It therefore appears that IAD and drug addiction are associated with, to some extent, similar corticostriatal functional circuits abnormalities in the brain, which may constitute a neural signature for these forms of addiction.

## Limitations

There are several limitations that should be mentioned in this study. Firstly, the diagnosis of IAD was mainly based on results of self-reported questionnaires, which may potentially result in error classification in some cases. Therefore, the diagnosis of IAD needs to be refined with standardized diagnostic tools to improve the reliability and validity. Secondly, the sample size in the study was relatively small and generalization of the findings should also be cautious. Owing to this limitation, the results should be considered preliminary and need to be replicated in future studies with a larger sample size. Thirdly, we exclude cases that were comorbid with substance and other major psychiatric disorders, and the results should be generalized cautiously to these groups with comorbid drug abuse and other psychiatric diseases. Fourthly, details of the duration of illness were not recorded in this study. Therefore, any association between the deficits in corticostriatal functional circuits and the duration of IAD could not be confirmed in this study. Fifthly, owing to limited resolution resting-state fMRI data, we examine FC based on a small number of subregions in the striatum, which may lead to incomplete depiction of the corticostriatal functional circuits. Therefore, high resolution resting-state fMRI data needed to be used to solve this problem in future studies. Lastly, without prospective investigations, the causal relationships between dysfunctions of the corticostriatal functional circuits and IAD could not be answered in this study. Future studies should attempt to identify the causal relations between IAD and the altered corticostriatal functional pathways.

## Conclusion

In summary, we used resting-state FC analysis to investigate the corticostriatal functional architecture in IAD adolescents. The results demonstrate that IAD is characterized by impairment of corticostriatal functional circuits involving affective and emotional processing, and cognitive control. The findings suggest that IAD may share psychological and neural mechanisms with other types of impulse control disorders and substance addiction. In addition, the associations between the connectivity strength of the corticostriatal circuits and behavioral measures indicate that corticostriatal circuits may serve as a potential new treatment target for IAD, and corticostriatal FC may be valuable in providing information on prognosis for IAD. Our results indicate that the abnormal resting-state corticostriatal FC may serve as an *in vivo* biomarker for testing new, potentially more effective, Internet addiction therapeutics.

## Author Contributions

FL, YZ, YD, JX and HL were responsible for the study concept and design. YZ, LQ and ZZ contributed to the acquisition of data. FL assisted with data analysis and interpretation of findings. FL drafted the manuscript. FL and HL provided critical revision of the manuscript for important intellectual content. All authors critically reviewed content and approved final version for publication.

## Conflict of Interest Statement

The authors declare that the research was conducted in the absence of any commercial or financial relationships that could be construed as a potential conflict of interest.

## Footnotes

^1^http://www.fil.ion.ucl.ac.uk/spm

^2^http://afni.nimh.nih.gov/pub/dist/doc/manual/AlphaSim.pdf
